# Establishment of “Structure‐Efficiency” Relationship in Ultra‐High Purity Metal Systems: Multi‐Scale Analysis of Tellurium as a Prototype

**DOI:** 10.1002/advs.202508531

**Published:** 2025-07-29

**Authors:** Shuai Guo, Xianglei Dong, Lin Zheng, Ming Gao, Guoqin Cao, Ping Peng, Jilin He, Junhua Hu

**Affiliations:** ^1^ School of Materials Science and Engineering Zhengzhou University Zhengzhou 450001 China; ^2^ National Key Laboratory of Special Rare Metal Materials Zhengzhou University Zhengzhou 450001 China; ^3^ State Center for International Cooperation on Designer Low‐Carbon & Environmental Materials (CDLCEM) Zhengzhou University Zhengzhou 450001 China; ^4^ CNBM (Chengdu) Optoelectronic Materials CO. Ltd Chengdu 610207 China; ^5^ School of Computational Science and Electronics Hunan Institute of Engineering Xiangtan 411104 China; ^6^ School of Materials Science and Engineering Hunan University Changsha 410083 China

**Keywords:** cross‐scale methodology, density functional theory, impurity separation efficiency, phase‐field simulations, structural evolution

## Abstract

As critical strategic materials, ultra‐high‐purity scattered metals play roles across cutting‐edge technological domains. Significant challenges remain in investigating the spatial location and chemical environment of impurities in high‐purity systems due to the limitations of conventional thermodynamic techniques and characterization resolution, hindering the improvement of purification efficiency. In this study, using tellurium as a model, a cross‐scale methodology is developed to elucidate the correlation between structural evolution and impurity separation efficiency. Experimental results show that increasing fusion rates induced transitions in growth orientation from (104) to (012) with reduced impurity content, accompanied by the morphological evolution from irregular to columnar grains. Phase‐field simulations reveal that the interface structure and grain competition drove the orientation transitions from (001) to (012). Density functional theory calculations confirmed the thermodynamic superiority of the (012) orientation, demonstrating weaker impurity adsorption at liquid / (012) interfaces versus liquid / (104). Based on these mechanisms, the Bridgman method is employed to enhance the preferred orientation in the tellurium crystal, significantly improving its purification efficiency. This multi‐scale investigation establishes a comprehensive framework for understanding the “structure‐efficiency” relationship in ultra‐high‐purity metals, providing theoretical guidance for the development of targeted deep purification technologies.

## Graphical abstract

By combining theoretical calculations with zone refining experiments, the quantitative relationship between purification efficiency and microstructure can be established. This multi‐scale investigation establishes a comprehensive framework for understanding the “structure‐efficiency” relationship in ultra‐high‐purity metals, providing theoretical guidance for the development of targeted deep purification technologies.

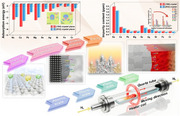

## 1 Introduction

Amid the rapid development of emerging industries, ultra‐high‐purity scattered metals with unique physical and chemical properties hold strategic significance in cutting‐edge fields such as electronic information, military applications, and aerospace technology.^[^ ^1^ ^]^ Against the backdrop of rapid technological advancements, the exponential growth in industrial applications has given rise to mounting demands for higher‐purity metal materials.^[^ ^2^ ^]^ Currently, investigations of ultra‐high‐purity metals mainly focus on optimizing purification processes and macroscopic separation efficiency.^[^ ^3^ ^]^ The production of ultra‐high‐purity materials faces significant challenges, including technical and economic. Especially, the extremely low yield of purification processes severely limits production efficiency, resulting in diminished technical feasibility and economic viability, directly contributing to the prohibitively high costs of ultra‐high‐purity metal. Furthermore, the precise characterization of impurity location and chemical environment during separation processes presents substantial scientific and technical hurdles, particularly in atomic‐level resolution. These limitations collectively constitute a major barrier to the further development of efficient deep purification technologies.^[^ ^4^ ^]^

When describing the occurrence state of impurities, it is essential to include four key aspects of impurities: who (impurity element), how many (quantity or concentration), where (location or distribution), and what the chemical state (environment or its interaction with host metal) it is. We heavily rely on high‐precision analytical instruments to obtain these data. However, inherent analytical constraints persist. For instance, inductively coupled plasma mass spectrometry (ICP‐MS) enables the quantitative detection of ultra‐low impurity concentrations but cannot reveal their spatial distribution or occurrence states.^[^ ^5^ ^]^ Aberration‐corrected transmission electron microscopy (AC‐TEM) can achieve visualization of impurity distributions in localized regions, but it offers only limited quantitative analysis.^[^ ^6^ ^]^ It is quite difficult to search for impurity atoms in an ultra‐diluted solid solution by TEM. Atomic probe tomography (APT) can precisely analyze the occurrence states of impurity elements in grain boundaries (GBs) or precipitated phases. Yet, its limited analysis area restricts its ability to reflect the overall sample purity.^[^ ^7^ ^]^ Additionally, the analytical limit of this methodology is insufficient to attain the ultra‐high‐purity levels. In traditional thermodynamic equilibrium phase diagrams, the boundaries between the liquid and solid phases converge as the solute concentration approaches zero. This limits their ability to control separation processes in ultra‐high‐purity systems. Besides, some impurity separation theories are derived primarily from low‐purity alloy systems, lacking accurate depictions of the occurrence states and interactions of trace impurities in ultra‐pure systems.^[^ ^8^ ^]^ These limitations impede in‐depth research on ultra‐high‐purity systems and obstruct a comprehensive understanding of the occurrence state and separation behavior of impurities.

Notably, the microstructure of the solid‐liquid interface is pivotal in dictating impurity distribution and separation behaviors.^[^ ^9^ ^]^ According to classical materials science theory, material properties are inherently dictated by structural characteristics spanning from atomic to macroscopic scales. Given this, studies on impurity behavior have garnered significant research interest, focusing on aspects such as solidified structural morphology, GBs, and their correlation with the spatial distribution of impurity elements, as well as segregation mechanisms.^[^ ^10^ ^]^ For example, Xing et al.^[^ ^11^ ^]^ revealed that GBs in irregular crystal morphologies obstruct impurity diffusion and transport, leading to impurity atom enrichment at GBs with specific configurations. Besides, Uda et al.^[^ ^12^ ^]^ demonstrated that the formation of grooves was the cause of impurity accumulation at GBs during solidification. Despite these insights, most studies of impurity separation have primarily focused on macro‐level industrial results, leaving unclear the mechanisms by which the microstructure affects impurity migration. In particular, systematic investigations into impurity behaviors at the atomic scale remain scarce.^[^ ^13^ ^]^ Thus, establishing the relationship between microstructure and separation efficiency is expected to provide a new research paradigm for the precise removal of impurities in ultra‐high‐purity metals.

In this work, tellurium (Te) was employed as a prototype to provide a multi‐scale interpretation for the impurity separation mechanism in the zone refining process. Experimentally, we systematically investigated the microstructural evolution controlled by the fusion rates. Especially, the increasing fusion rate induced the preferred oriented columnar crystal, significantly reducing the impurity concentration. Phase‐field simulations revealed the competitive dynamic mechanism of different orientations, driven by the interface structure and grain competition. Density functional theory (DFT) calculations quantified the differences in adsorption energies of impurity atoms on various crystal planes at the atomic scale, elucidating the mechanism of interfacial selective impurity separation. A multi‐scale modeling approach was proposed to establish the “structure‐property” relationship. The different dependences of the impurity occurrence state on the microstructure can be used for intelligent purification with high economic effectiveness.

## 2 Results and Discussion

### 2.1 Structure‐Efficiency Relationships Between Purification Efficiency and Microstructure Evolution

**Figure 1:**
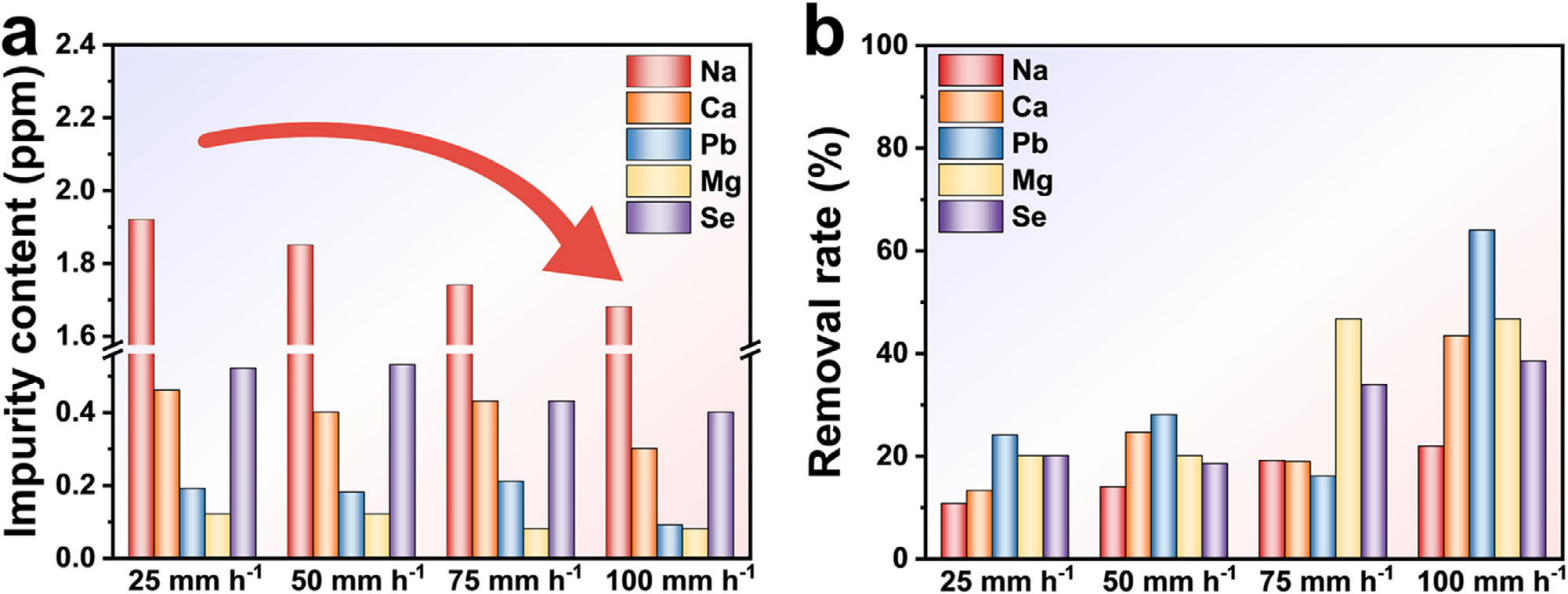
Impurity analysis of samples at different fusion rates. a) Impurity content. b) Impurity removal rate.

We adopted ICP‐MS to analyze the impurity content in samples at different fusion rates (the error margin is less than ±5%) and compared the results with those of the raw materials to obtain the impurity removal rate.^[^ ^14^ ^]^ As shown in **Figure** **1**a, the impurity content of Na, Ca, Pb, Mg, and Se decreased with increasing fusion rates. Correspondingly, the impurity removal rate exhibited an increasing trend (Figure 1b). Especially, the increased fusion rates can dramatically shorten impurity diffusion time and distance, suppressing their interfacial enrichment while enhancing impurity separation efficiency.^[^ ^15^ ^]^ In another aspect, the purification efficiency of other impurity elements displayed a random tendency with fusion rates, as shown in Figure S1 (Supporting Information). The complex dependence of removal efficiency on the fusion rate was reported by many studies, which could not be interpreted only by impurity diffusion.^[^ ^16^ ^]^ Accordingly, the morphological evolution of solid‐liquid interfaces may govern the differential purification behaviors among impurity elements.

**Figure 2:**
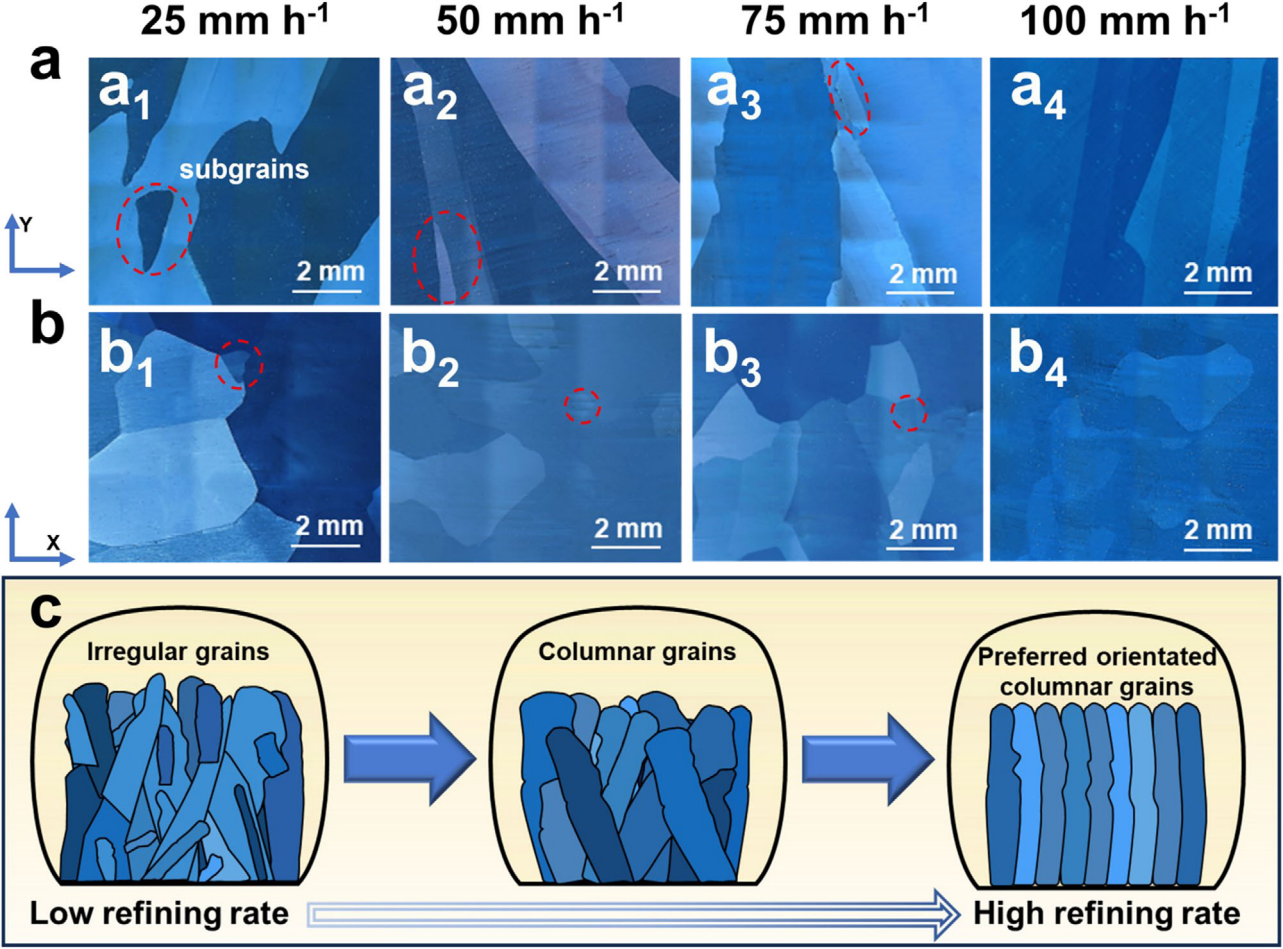
Microscopic morphology analysis. a,b) Metallographic images of samples: the sample section in (a_1_‐a_4_) Y‐direction and (b_1_‐b_4_) X‐direction. c) Schematic diagram of crystal structure transformation.

The effects of varying fusion rates on microstructural characteristics and crystallographic orientation were analyzed to explore the relationship between impurity separation and structure evolution. As illustrated in **Figure** **2**a,b, the Te sample at 25 mm h^−1^ exhibited irregular grains with minor subgrains in both the Y and X directions. With an increasing fusion rate, homogeneously oriented columnar grains were formed in the Y direction at 100 mm h^−1^. Consequently, irregular grains gradually transitioned into columnar grains as fusion rates increased, forming preferentially oriented columnar grains at a high fusion rate (Figure 2c).

**Figure 3:**
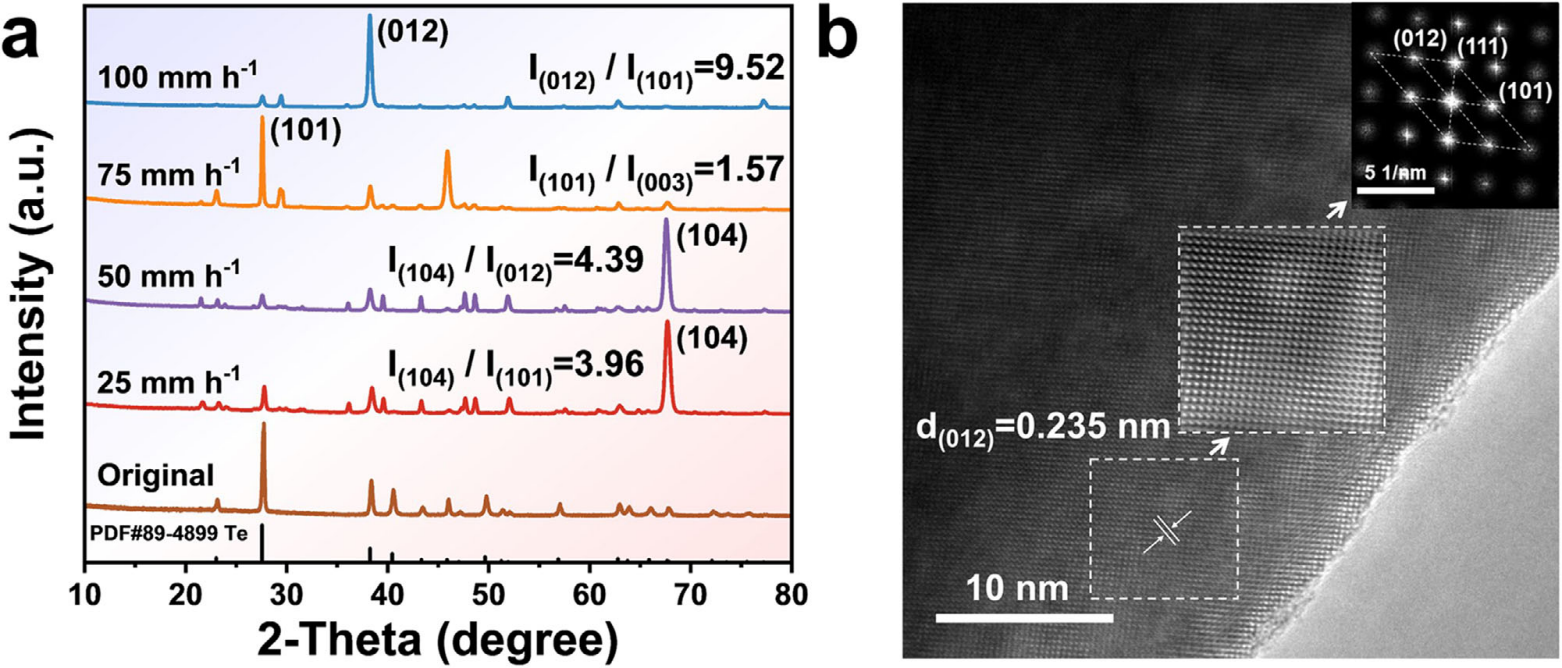
Structural characterization and crystal plane orientation analysis. a) XRD patterns of the specimens with different zone fusion rates. b) HRTEM and corresponding FFT images of the sample at 100 mm h^−1^.

As shown in **Figure** **3**a, X‐ray diffraction (XRD) was employed to examine the growth orientation of Te crystals under different fusion rates. The original powder material was analyzed for comparison, corresponding to the standard diffraction file PDF#89‐4899. Progressive crystallographic orientation transitions were observed with the increasing fusion rates. At 25 and 50 mm h^−1^, the strongest diffraction peak at 67° corresponded to the (104) crystal plane. As the fusion rate reached 75 mm h^−1^, the dominant orientation shifted to (101), aligning with the original material (raw Te materials). Upon reaching 100 mm h^−1^, the specimen exhibited a pronounced (012) preferred orientation. The ratio of the primary peak intensity to the secondary peak intensity was defined as an indicator of the degree of preferred orientation. Specifically, the *I_(012)_* / *I_(101)_* at 100 mm h^−1^ reached 9.52, demonstrating a significantly higher degree of preferential orientation compared to the *I_(104)_* / *I_(101)_* ratio of 3.94 at 25 mm h^−1^. This ratio markedly exceeded the range of 1‐2.5 reported in others, indicating that our samples achieved a unique degree of preferential orientation (Table S2, Supporting Information). In addition, the HRTEM lattice image and the corresponding fast Fourier transform (FFT) pattern in Figure 3b confirmed high crystallinity, defect‐free structure, and preferred growth of the Te (012) plane at 100 mm h^−1^.

### 2.2 Orientation Selection Mechanisms of the Te Grains

To further elucidate the orientation selection mechanisms of Te grains during deep impurity separation. A multi‐phase field model was employed to simulate the interface evolution and impurity segregation in zone refining.^[^ ^17^ ^]^ In the phase field model, a series of order parameters (*α* = 1, 2, …, *s*, *l*) was used to quantify the local phase fraction for crystal orientation of *α*, where *s* is denoted by the number of crystal orientations and *l* is the liquid phase. *ϕ_α_* = 1 represents the pure solid phase with orientation of *α* while *ϕ_α_* = 0 represents the liquid phase or solid phase with other orientations, and the value of *ϕ_α_* continually changes from 0 to 1, representing the liquid‐solid interface. Furthermore, a series of dimensionless parameters *u_i_* (*i* = 1, 2, …, *n*) was used to estimate the local impurity composition for component of *i* during the impurity separation process, where *n* was denoted by the total number of impurities. *u_i_* was satisfied by,

(1) $$\begin{array}{c} 1+\left(1-k_{i}\right)u_{i}=c_{i}/\left\{{c_{i}}_{l}^{0}\left[1-\left(1-k_{i}\right)\sum_{\alpha =1}^{s}\phi_{\alpha }\right]\right\} \end{array}$$

where *c_i_* and ${c_{i}}_{l}^{0}$ are actual and nominal compositions in front of the solid‐liquid interface, respectively, and *k_i_* is the equilibrium segregation coefficient. In the multi‐phase‐field model, evolutions of *ϕ_α_* and *u_i_* can be described by,

(2) τ0∑iDl,refDl,refDl,i·Dl,i·1+1−kiuiMias2n⃗α∂tϕα=W02∇·as2(n⃗α)∇ϕα+W02∂x|∇ϕα|2as(n⃗α)∂as(n⃗α)∂∂xϕα+W02∂y|∇ϕα|2as(n⃗α)∂as(n⃗α)∂∂yϕα−fϕα−λgϕα∑i=1nMiui+Gx−VptGx−Vptmi(1−ki)cli0mi(1−ki)cli0

(3) $$\begin{array}{cc} & \left[1-\left(1-k_{i}\right)\sum_{\alpha =1}^{s}\phi_{\alpha }\right]\partial_{t}u_{i}\\ & \;=\nabla \cdot \left\{D_{i}\left(\phi_{\alpha }\right)\nabla u_{i}-a_{i}W_{0}\left[1+\left(1-k_{i}\right)u_{i}\right]\sum_{\alpha =1}^{s}\partial_{t}\phi_{\alpha }{\vec{n}}_{\alpha }\right\}\\ & \;+\left[1+\left(1-k_{i}\right)u_{i}\right]\sum_{\alpha =1}^{s}\partial_{t}\phi_{\alpha }\\ & \;+\left[1+\left(1-k_{i}\right)u_{i}\right]\sum_{\alpha =1}^{s}\partial_{t}\phi_{\alpha } \end{array}$$

Equation (2) and (3) were called the phase‐field equation and the solute field equation, respectively. In these equations, *G* is the temperature gradient, *V_p_* is the pulling velocity, *m_i_* is the slope of the liquid phase line, λ is the coupling coefficient and n⃗α=−∇ϕα/−∇ϕα|∇ϕα||∇ϕα| is the unit normal vector perpendicular to the interface. *M_i_* = *d*_0_/*d*_0*i*_ is a series of weight factors, which was used to estimate the undercooling capacity by considering the individual impurity segregation, where *d*_0*i*_ is the capillary length for component of *i*, and $d_{0}=1/\sum_{i=1}^{n}\left(1/d_{0i}\right)$ is defined as the effective capillary length for the total purification system.$f_{\phi_{\alpha }}=2\phi_{\alpha }\left(1-\phi_{\alpha }\right)\left(1-2\phi_{\alpha }\right)$ and $g_{\phi_{\alpha }}=\phi_{\alpha }^{2}{\left(1-\phi_{\alpha }\right)}^{2}$ are free energy densities related to the phase transition. $D_{i}\left(\phi_{\alpha }\right)=D_{li}\left(1-\sum_{\alpha =1}^{s}\phi_{\alpha }\right)+D_{si}\sum_{\alpha =1}^{s}\phi_{\alpha }$ is an interpolation function related to the impurity diffusion, where *D_li_* and *D_si_* are diffusion coefficients for component of *i*, and *D*_*l*,*ref*_ is a reference diffusion coefficient with *D*_*l*,*ref*_ = *D*_*l*,*Sn*_in this work. *W*_0_ and *τ*_0_ are spatial scale and time scale related parameters, which were used to characterize the thickness of the diffusion interface and the dynamic migration of the interface, respectively. According to the quantitative asymptotic analysis, *W*_0_ and *τ*_0_ were satisfied with *d*_0_ = *a*_1_ *W*_0_/λ and $\tau_{0}=a_{2}\lambda W_{0}^{2}/D_{l,ref}$, which established the quantitative relationship between the diffused interface model and the practical process.

During the simulation, the pure Te has a six‐fold symmetric hexagonal structure, and the energy anisotropy distribution of the solid‐liquid interface was defined as:

(4) $$a_{S}\left(\vec{n}\right)=1+\varepsilon_{20}Y_{20}\left(\theta ,\varphi \right)+\varepsilon_{66}Y_{66}\left(\theta ,\varphi \right)$$

where *ε_20_* and *ε_66_* are the anisotropy coefficients, reflecting the interface energy contribution on the cylindrical and basal plane of the hexagonal structure, respectively.^[^ ^18^ ^]^ Here, we used *ε_20_* = ‐0.104, *ε_66_* = 0.016, which was proved to be suitable for the hexagonal structure.^[^ ^19^ ^]^ It is assumed that the crystal planes (10*k*) were equivalent to (01*k*) in the mesoscale model, where *k* = 0 means the cylindrical plane, *k* = 2 means the pyramidal plane, and *k*→∞ means the basal plane (001).

**Figure 4:**
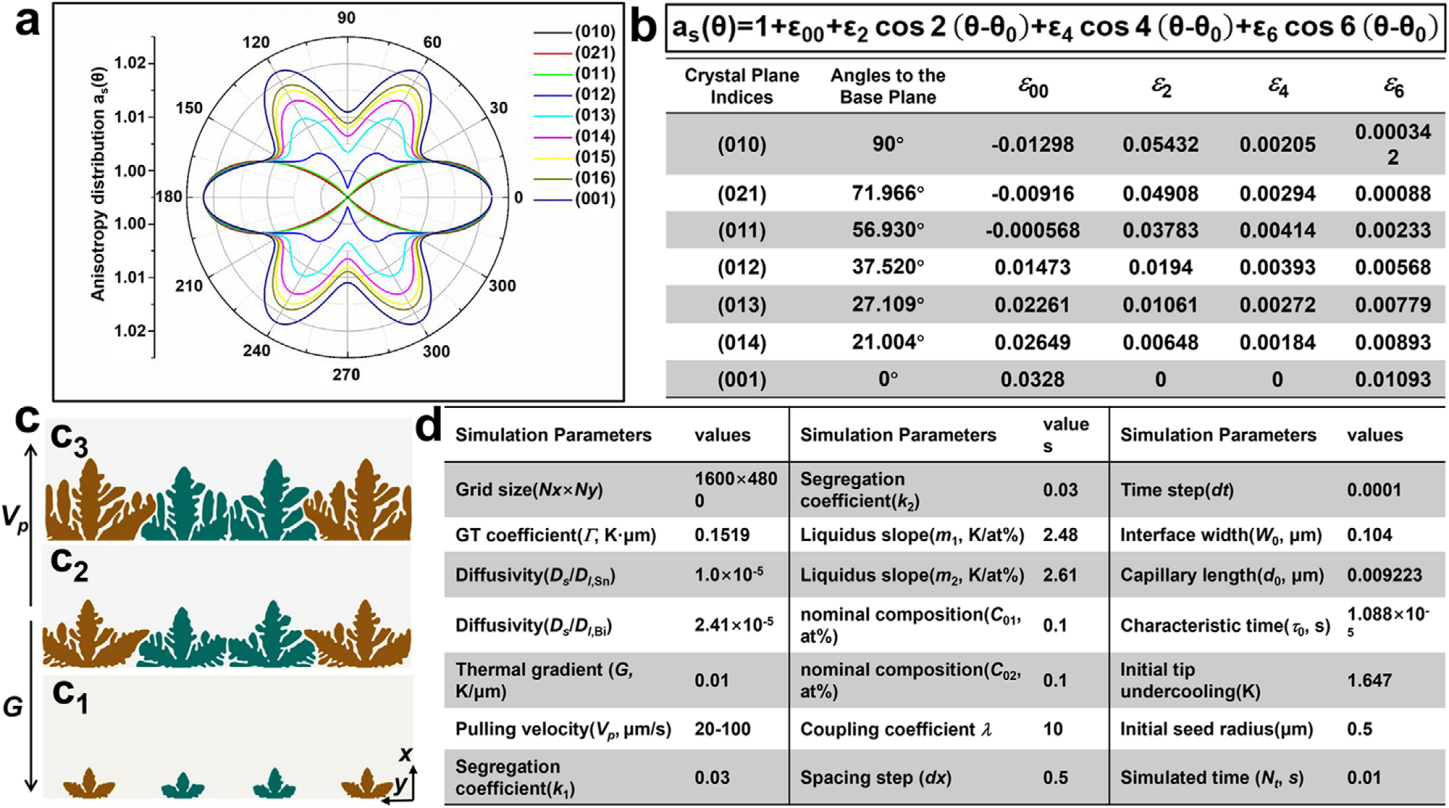
The initial configuration of the phase‐field simulation. a) Polar distribution of the anisotropy interface energy for different crystal planes. b) Table of anisotropy coefficients for different crystal planes calculated by Fourier analysis. c) Initial interface patterns of Te dendrites with Sn, Bi impurities with the dimensionless simulated time of *t* / *τ*_0_ = 10 (c_1_), 20 (c_2_), 30 (c_3_) for the (011) (brown) and (012) (Green) crystal planes. d) Table of model or materials parameters for the phase‐field simulation of competitive growth in the micro alloyed Te‐0.1%Sn‐0.1%Bi system.

**Figure** **4**a illustrates the anisotropy of interface energy, where different crystal planes were projected into the identical (001) plane. All these (01*k*) planes possessed the preferred orientation along the *x* direction, with nearly identical anisotropy distribution within the interval of [−30°, 30°]. At the direction of *θ* = 60°, however, the orientation preference weakened as the *k* decreased in the planes (01*k*), where the preferred orientation of *θ* = 60° was absent at *k* ∈ [0, 1]. Although it was limited to a 2D projective plane, the anisotropy distribution presented a good description of interface structure for differently oriented crystal planes at a mesoscopic scale. We aim to further investigate the growth competition of differently oriented crystals when the pulling velocity (*V_p_*) and thermal gradient (*G*) were parallel to the basal plane and along the *x* direction. In this case, it would be clear which plane of the (01*k*) family was most preferred, as they exhibited energy anisotropy. Considering that the symmetry of (01*k*) planes is 2‐fold or 6‐fold, a 2D anisotropy function can be employed to quantitatively describe the interface energy distribution by using Fourier analysis. The new anisotropy function and its coefficients were listed in Figure 4b, making it possible to carry out the 2D simulation of columnar growth competition among different crystal plane orientations. Here, two cases were selected to study the growth competition: (1) The (012) plane versus the (011) plane (abbreviated as (012)‐(011)); (2) The (012) plane versus the (001) plane (abbreviated as (012)‐(001)). The first case corresponds to (012)‐(101) oriented competition in the experiment, while the second case corresponds to (012)‐(104) oriented competition in the experiment. Figure 4c shows the initial configuration and the interface evolution in the case of (012)‐(011) with symmetric boundary conditions along both the *x* and *y* directions. A system of Te containing Sn and Bi impurities was used in the simulation, with the model and material parameters listed in Figure 4d. Although the database is insufficient for rare and scattered metals, we adopted certain material parameters based on the dilute alloy approximation. Moreover, thin interface analysis and convergence studies were carried out to establish the quantitative relationships between the material parameters and model variables, which ensured the intrinsic discussion of the growth competition mechanisms for Te separation and purification.

**Figure 5:**
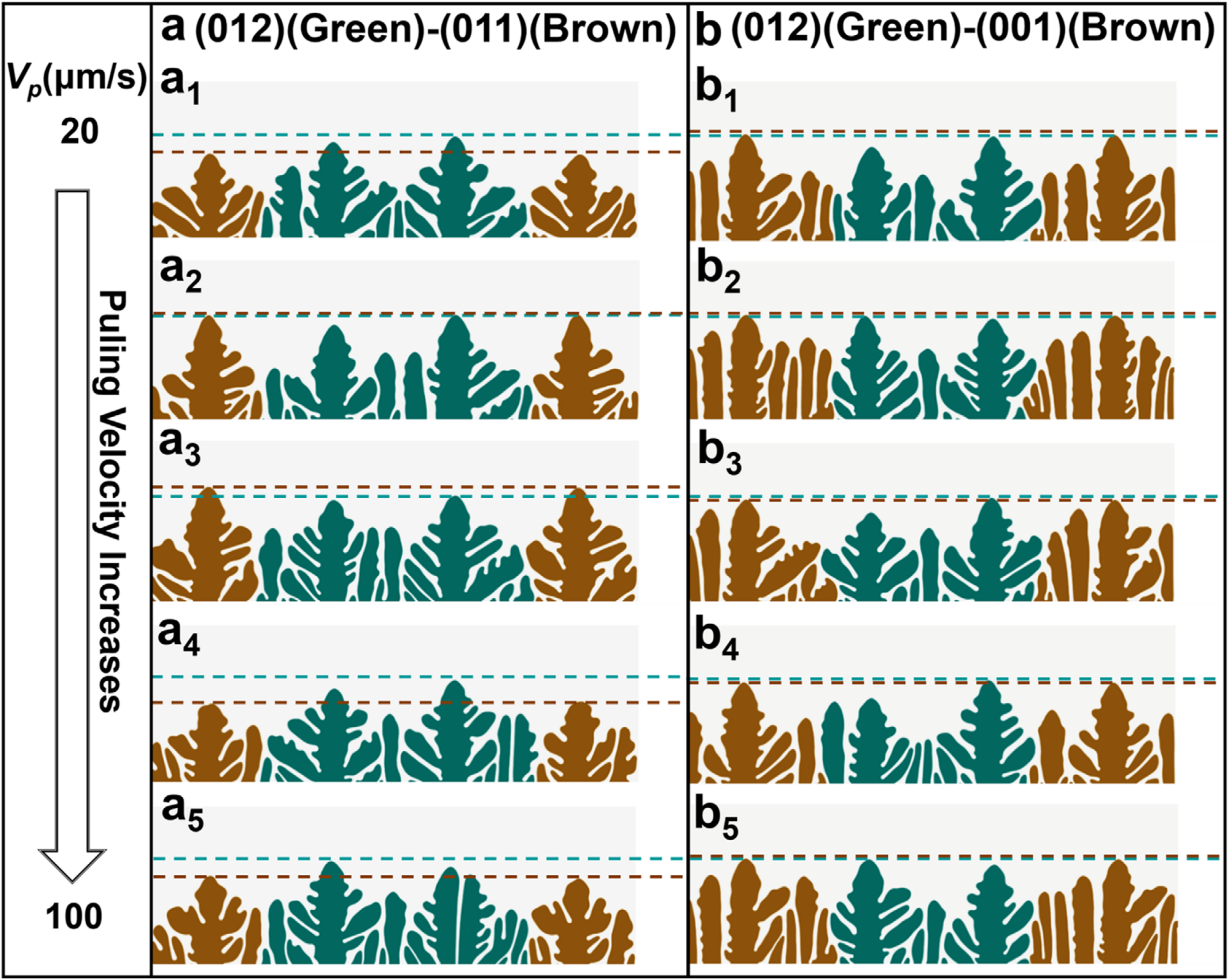
The competitive dendrite patterns for a long‐period simulation in the (012)‐(011). a) Competitive growth patterns in the case of (012) (Green)‐(011) (Brown) by increasing the pulling rate of *V_p_* = 20 (a_1_), 40 (a_2_), 60 (a_3_), 80 (a_4_), and 100µm s^−1^ (a_5_) for a long simulated time of *t* / *τ*_0_ = 900. b) Competitive growth patterns in the case of (012) (Green)‐(001) (Brown) by increasing the pulling rate of *V_p_* = 20 (b_1_), 40 (b_2_), 60 (b_3_), 80 (b_4_), and 100µm s^−1^ (b_5_) for a long simulated time of *t* / *τ*_0_ = 900.

**Figure** **5**a shows the competitive dendrite patterns for a long‐period simulation in the (012)‐(011). It was observed that the (012) dendrites grew ahead of the (011) dendrites at the lower fusion rate (*V_p_* = 20µm s^−1^). As *V_p_* increased, the (012) dendrites started to fall behind the (011) dendrites, typically at a rate of 60µm s^−1^. With a further increase in *V_p_*, the (012) dendrites became the leading orientation again during the growth competition. In the case of (012)‐(001) competitive growth, the leading orientations with different velocities are hardly recognizable from Figure 5b, suggesting that the competitive behavior is more significant for (012)‐(011) planes compared with (012)‐(001) planes.

**Figure 6:**
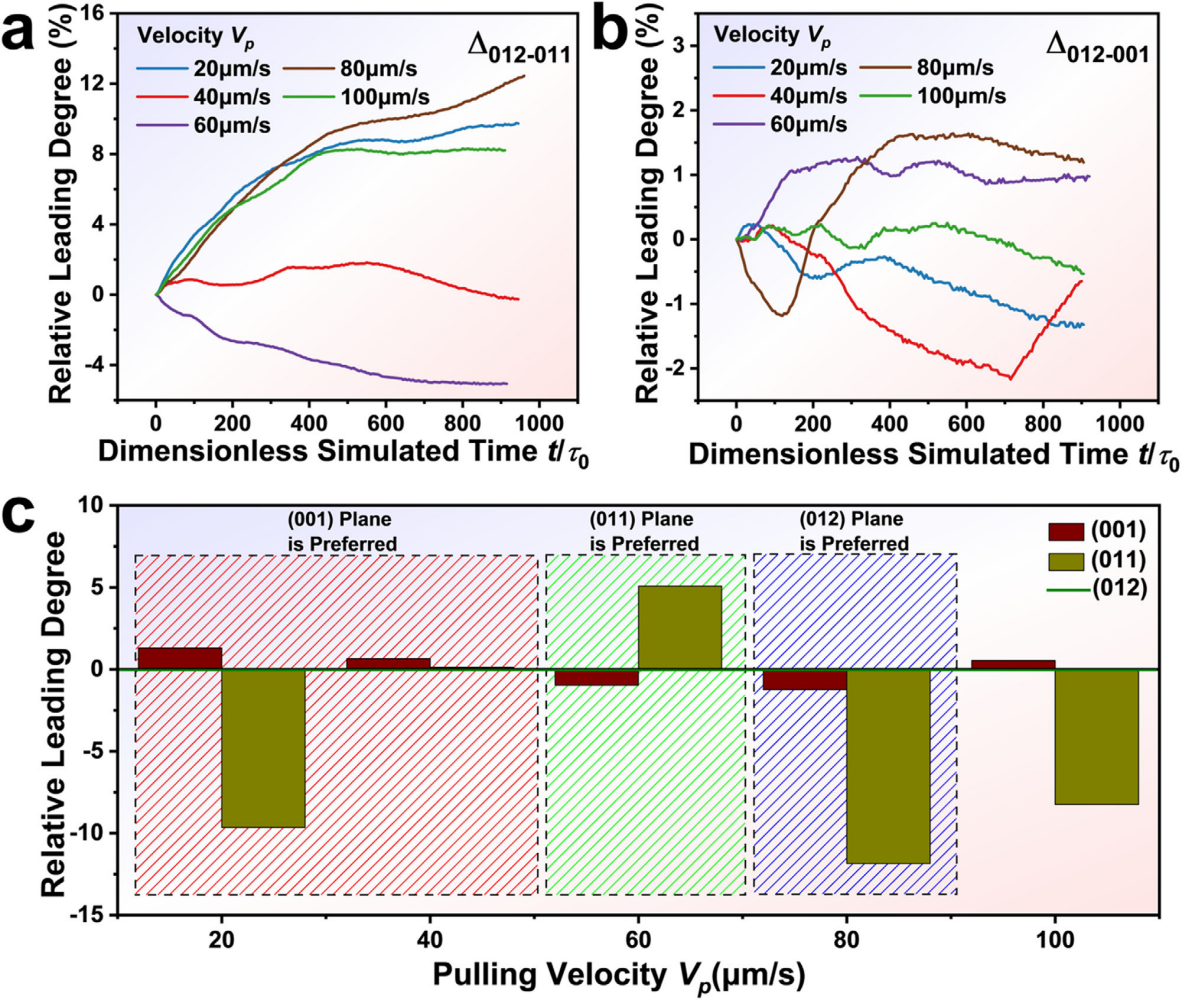
Quantitative analysis of the growth competition. a) The relative leading degree *Δ_012‐011_* as a function of the dimensionless simulated time *t* / *τ*_0_ with the different pulling velocities. b) The relative leading degree *Δ_012‐001_* as a function of the dimensionless simulated time *t*/*τ*_0_ with the different pulling velocities. c) Relative leading degree of (001) (Wine Column)‐(012) (Green Line)‐(011) (Yellow Column).

To provide a quantitative analysis of the growth competition, the relative leading degree (RLD) was introduced to quantify the difference in tip undercooling between two competitive planes. Δ_012‐001_ was defined as 2 (Δ_012_ ‐ Δ_001_) / (Δ_012_ + Δ_001_) × 100%, while Δ_012‐011_ was defined as 2 (Δ_012_ ‐ Δ_011_) / (Δ_012_ + Δ_011_) × 100%, where Δ_012_, Δ_011_ and Δ_001_ were the undercooling of the (012), (011), and (001) dendritic tips, respectively. **Figure** **6**a,b illustrates the time evolution of RLD as a function of fusion rate for (012)‐(011) and (012)‐(001), respectively.

The competition mechanism of (012)‐(011) can be understood by analyzing the side‐branching behavior between dendrites. At a lower fusion rate of *V_p_* = 20µm s^−1^, the (012) dendrite exhibited more abundant side branches than the (011) due to the interface energy anisotropy. The (012) plane had a secondary preferred orientation *θ* = 60° (as shown in Figure 4a), which was absent in the (011) plane, driving side‐branches to compete with the (012) primary dendrites. However, due to the low undercooling, tertiary side branches were blocked by secondary side branches of two neighboring (012) primary dendrites, and thus their growth was more facilitated near the GBs. The segregation of the tertiary side branching suppressed the impurity diffusion from the (011) dendrite to the liquid. Hence, (012) dendrites grew ahead of (011) dendrites. When the fusion rate increased to 40‐60 µm s^−1^, the growth of tertiary side branches between (012) dendrites was enhanced, accelerating impurity rejection in the inter‐dendritic region. In this case, tertiary side branches hindered the normal impurity diffusion of secondary arms or even the primary dendrites, resulting in the (011) dendrite becoming dominant. At a higher rate of *V_p_* 80–100 µm s^−1^, the undercooling difference between primary and side‐branching dendrites was sufficiently large that tertiary side branches no longer influenced the primary dendrite growth. Meanwhile, due to surface energy anisotropy, the (012) tertiary side branches gained a significant competitive advantage over the (011) branches at the GBs. Overall, the (012) dendrites regained their leading position at higher fusion rates due to the combined effects of impurity diffusion and interface energy anisotropy.

A reasonable interpretation of the competitive behavior between (012) and (001) planes was shown in the curves of Figure 6c. At a lower fusion rate of *V_p_* = 20–40 µm s^−1^, the (001) dendrites slightly outgrew (012) ones, due to their more preferred orientation *θ* = 60°, which was attributed to an interface structure‐induced orientation competition mechanism. As the fusion rate increased to 60‐80 µm s^−1^, the increased undercooling intensified inter‐dendritic competition between primary dendrites and side branches, causing the (001) dendrites to lag behind the (012). At a higher fusion rate of *V_p_* = 100 µm s^−1^, the increased driving force minimized the impact of side branch growth on primary dendrites, allowing the (001) dendrites to once again outgrow the (012) ones. This phenomenon can be attributed to the combined effects of impurity diffusion and interface structure‐driven orientation competition mechanisms. Considering both cases of dendritic growth competition, the orientation transition can be understood as a transition from the preferred (001) plane to (011), and ultimately to (012). Notably, the observed transition trend aligns well with zone refining experiments ((104) to (101), and ultimately to (012)). Therefore, we believe that the combined effects of impurity diffusion and interface structure play a crucial role in elucidating the orientation competition mechanisms governing impurity separation in the zone refining process.

Additionally, according to the competitive grain growth model proposed by Walton and Chalmers, the competitive growth behavior of grains was governed by undercooling ahead of the solid‐liquid interface, as illustrated in Figure S2 (Supporting Information).^[^ ^20^ ^]^ The undercooling difference between non‐preferred and preferred orientation grains was minimal at low fusion rates, leading to weak competition in crystal growth and irregular grain shapes.^[^ ^21^ ^]^ As the fusion rate increased, the undercooling difference gradually increased. In contrast, the preferred orientation grains gradually gained a competitive advantage and suppressed others, leading to the columnar grains with a preferred orientation. Consequently, irregular grains transitioned into columnar grains as the fusion rates increased and eventually transformed into preferred‐oriented columnar grains.

### 2.3 Atomic‐Scale Relationship of Crystal Orientation and Impurity Segregation

**Figure 7:**
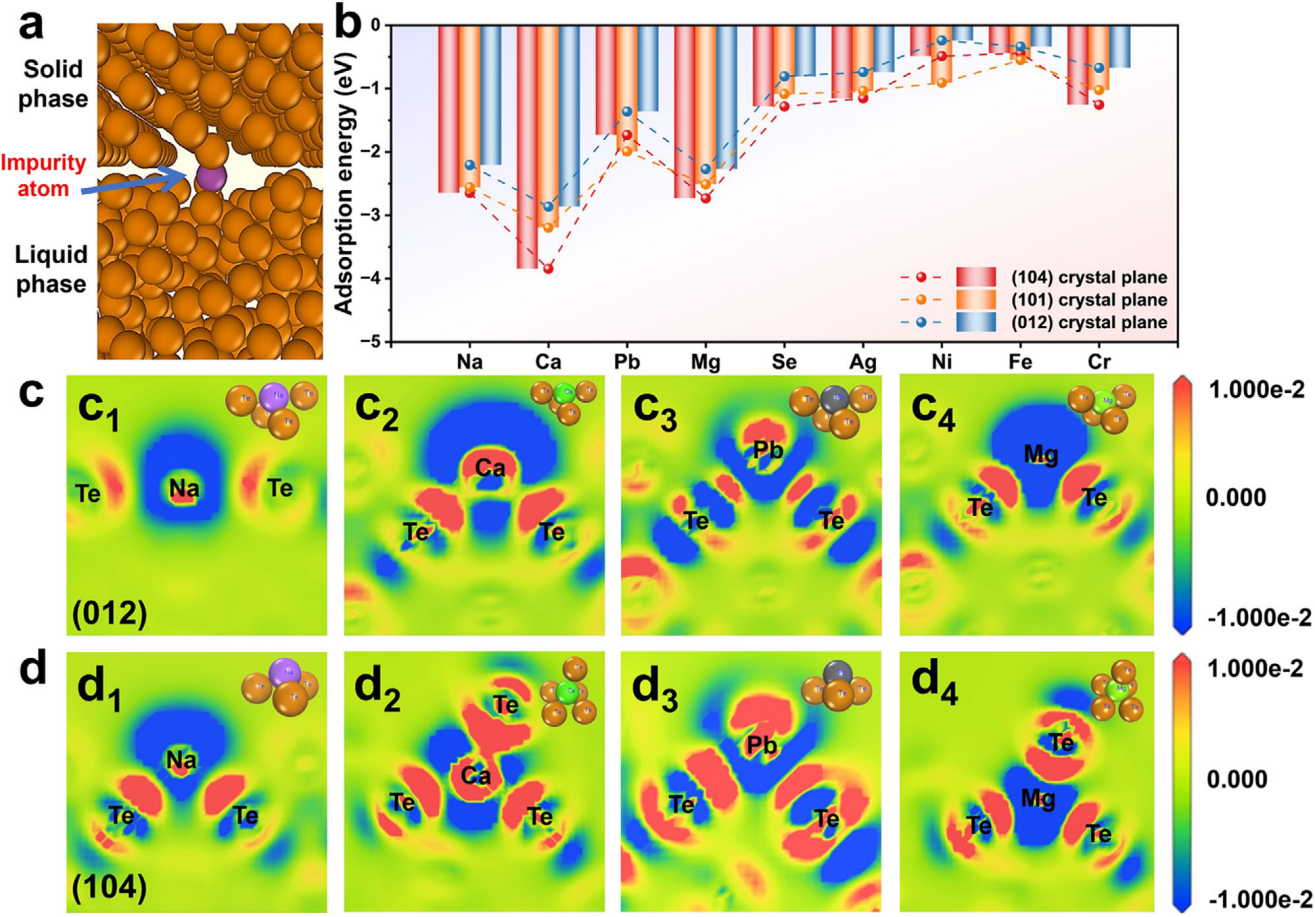
DFT calculations. a) Diagram of impurity atoms at the solid‐liquid interface of Te. b) Adsorption energies of Te on (104), (101), and (012) crystal planes for different impurity elements. c,d) Differential charge density maps of impurity elements on (c_1_‐c_4_) the (012) and (d_1_‐d_4_) the (104) crystal planes of Te.

In response to the mechanism proposed above, the adsorption energies of impurity atoms on the typical (012), (101), and (104) planes at the solid‐liquid interfaces were quantified to reveal the influence of the microstructure on the impurity segregation process (**Figure** **7**a,b). Here, adsorption energy was defined as the change in the system energy of impurity atoms that migrated from the solid‐liquid interface and became trapped by the solid phase. A lower adsorption energy value indicated a more favorable adsorption process, which was not conducive to impurity separation. Notably, the (012) plane exhibited higher adsorption energies for all investigated impurity elements than the (101) and (104) planes, suggesting weaker binding interactions at the solid‐liquid interface. In particular, the solid‐liquid interface exhibited lower adsorption energies for Na, Ca, Pb, and Mg impurities, which meant that the migration of these impurities was greatly affected by the adsorption of the solid‐liquid interface. In combination with Figure 1b, it was found that these impurity elements showed a significant removal rate enhancement trend with the evolution of (104) to (012) preferred orientation, demonstrating strong dependence on interface structure. Additionally, planes with higher surface energy were more conducive to the adsorption of impurity atoms, and those of (104), (101), and (012) were calculated to be 388.2, 264.3, and 169.3 mJ m^−2^, respectively (Figure S3, Supporting Information). This meant that the (012) crystal plane could suppress the adsorption of impurity atoms on the solid side, and further validated the clear structural‐activity relationship between crystal orientation and the efficiency of impurity separation.

Differential charge density analysis revealed the changes in charge distribution between atoms at the solid‐phase interface, where the red zone represented the charge accumulation regions and the blue zone represented the charge‐depleted regions (Figure 7c,d). Specifically, taking Na as an example, the charge accumulation between Te and Na atoms was weaker on the (012) plane than that on the (104) plane, indicating a weaker Na‐Te interaction on the (012) plane, which led to fewer impurity atoms remaining in the solid phase. A similar trend was observed for other impurities, such as Ca, Pb, and Mg, further highlighting the advantage of (012) preferred growth in promoting effective impurity separation.

### 2.4 Feasibility Validation of Orientation Selection for Facilitating Impurity Separation

**Figure 8:**
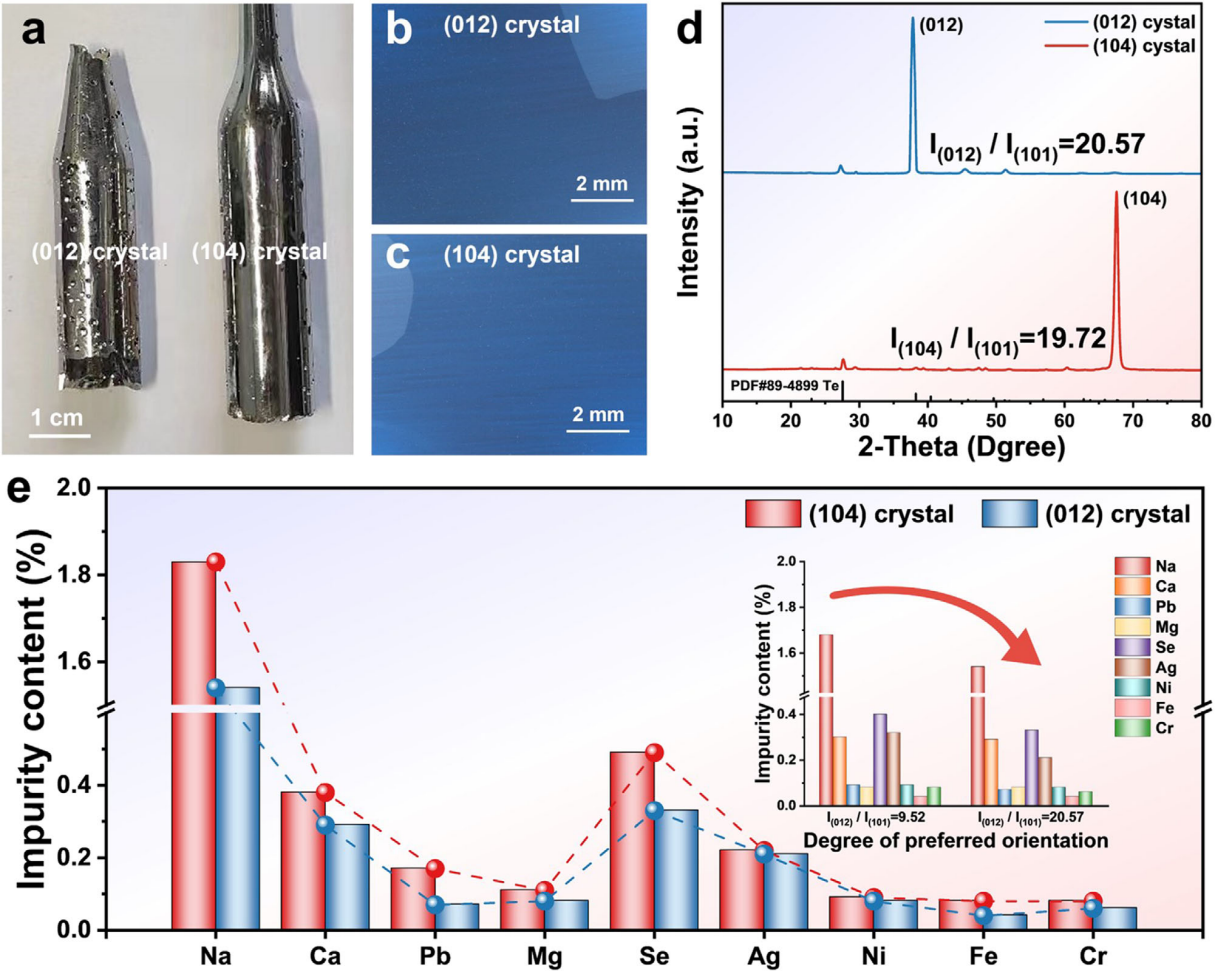
The crystals with (012) and (104) preferred orientation. a) optical photograph of Te crystals. b‐c) metallographic images. d) XRD patterns. e) impurity content.

To further validate the feasibility of promoting impurity separation through the preferred orientation selection, the Bridgman method was employed to fabricate Te crystals with higher preferred orientations, as shown in **Figure** **8**a,b. Compared with those in Figure 3a, the *I_(012)_* / *I_(101)_* = 9.52 and *I_(104)_* / *I_(101)_* = 3.96 were improved to 20.57 and 19.72, respectively (Figure 8b–d). As shown in Figure 8e, the purification results of samples were greatly affected by crystal plane orientation and the degree of preferred orientation. Especially, compared with the (104) oriented samples, the overall impurity removal rate of the (012) oriented samples increased from 27.4% to 42.9%, further confirming the significant influence of interface structural evolution on the deep separation of trace impurities.

This multi‐scale research method and the structure‐function relationship framework have the potential to be applied to other ultra‐high‐purity metal systems. By combining theoretical calculations with zone refining experiments, we can establish the quantitative link between purification processes and crystallographic plane or other microstructures, which will guide the improvement of purification efficiency as long as the interface microstructures can be precisely controlled.

## 3 Conclusion

This work aims to establish a “structure‐efficiency” relationship in a case study of Te purification through a synergistic multi‐scale computational‐experimental approach. The results indicated that impurity elements such as Na, Ca, Pb, and Mg showed a strong dependence on structure evolution. With increasing fusion rate, the preferred crystal orientation of Te transitioned from (104) to (101) and ultimately to the (012) plane. Correspondingly, the structural evolution mechanism was elucidated through phase‐field simulation, which highlighted the synergy between the interface structure and grain competition, inducing competition between primary‐side branches. From an atomic scale perspective, the adsorption of the impurity atoms at the liquid / (012) plane interface was weaker than that at other typical plane interfaces, which verified the advantage of (012) crystal orientation, benefiting the separation of trace impurities. A multi‐scale approach focusing on the “structure‐function” relationship between microstructure and impurity separation behavior offers a new perspective on material purification. More importantly, this novel approach demonstrates broad applicability, as it can be readily extended to other materials, revealing the complicated relations of interface structure, separation process, and purification efficiency in ultra‐high‐purity systems.

## 4 Experimental Section

### Zone Refining Experiments

This study employed commercially available 5N Te as the raw material. The impurities and their concentrations were analyzed by ICP‐MS, with the primary detected impurities listed in Table S2 (Supporting Information). To ensure accuracy, each sample was tested three times and averaged. The impurity elements in the sample included Se, Na, Ca, Pb, Mg, Ag, Ni, Fe, Cr, etc. The horizontal zone fusion equipment with industrial kilogram‐scale production capacity was utilized for purification (Figure S5a, Supporting Information). The bulk raw Te (3.5 kg) was placed in a quartz boat (550 mm × 50 mm × 50 mm) and loaded into the quartz tube of the zone refining setup. To prevent oxidation and contamination of the samples during the experiment, high‐purity hydrogen gas was continuously introduced to purge the air from the tube while ensuring air‐tightness. The apparatus operated in conjunction with an induction heater, and experiments were conducted by varying the moving speed of the heating coil to achieve different fusion rates (25, 50, 75, 100 mm h^−1^). The fusion length was 55 mm, and each experiment was conducted in one pass. Zone refining was a process of melting and crystallization, during which impurity atoms were redistributed at the solid‐liquid interface. The equilibrium distribution coefficients of all typical impurity elements were below 1, indicating their preferential segregation into the liquid phase.^[^ ^22^ ^]^ As the molten zone moved from the head to the tail of the sample, impurity atoms tended to accumulate at the tail, thereby achieving high purity (Figure S5b, Supporting Information). The samples were extracted from the middle for purity analysis. To observe the microstructural evolution at various positions, the sample section in both the *Y*‐direction and *X*‐direction was obtained for the morphology and structural characterization (Figure S5c, Supporting Information).

### Preparation of Te Crystals with (012) and (104) Preferred Orientation

Te crystal growth was carried out in the Bridgman furnace, as shown in Figure S6 (Supporting Information). The growth crucible containing raw Te was evacuated to 2×10^−5^ Pa, and the growth crucible was encapsulated with the hydroxide flame. The Te crystals were grown at 452°C with a temperature gradient of 15°C cm^−1^ and a growth rate of 1.5 mm h^−1^ by adjusting the heating zone. Seed crystals were cut from the crystal along the (012) and (104) crystal planes using the diamond wire cutter (STX‐202A, China). Then, the growth was induced by placing seed crystals in the seed crystal groove of the crucible. Finally, Te crystals with (012) and (104) preferred orientation were obtained.

### Material Characterizations

The content of each impurity element was analyzed by ICP‐MS (Agilent, 7800). The optical microscope (Zeiss, Axio Scope.A1) was used to compare the microstructural characteristics of samples with different fusion rates. The structural properties of samples were determined using XRD (Bruker, D8 Advance) and HRTEM (FEI, Tecnai F20).

### Computational methods

Dynamic equations in the multi‐phase‐field model were solved by the finite difference method. A specific solving algorithm was programmed by using the C++ language and the NVIDIA^®^ CUDA parallel computing technology. The programming environment was based on the Microsoft^®^ Visual Studio 2022 and Intel^®^ one API Toolkits. Programmed codes were compiled and executed by using a server with Intel^®^ Xeon 6133 × 2 CPUs for C++ hosts and NVIDIA^®^ TESLA V100 32GB × 8 GPUs for CUDA devices.

First‐principles calculations were conducted by using the DMOL3 software package based on Density Functional Theory (DFT).^[^ ^23^ ^]^ In the process of geometry optimization and total energy calculations, relativistically corrected Effective Core Potentials (ECP) pseudopotentials were employed, and the atomic wave functions used a double numerical basis set with d‐polarization functions.^[^ ^24^ ^]^ The exchange‐correlation potential was chosen as the Perdew‐Burke‐Ernzerhof (PBE) exchange‐correlation functional within the General Gradient Approximation (GGA).^[^ ^25^ ^]^ The energy deviation was kept below 1.0 × 10^−5^ Ha, stress changes below 0.002 Ha Å^−1^, and displacement deviations below 0.005 Å. The vacuum layer of the surface adsorption model was set to 16 Å, and the bottom layer atoms were fixed during structural optimization to allow atoms near the surface to relax, ensuring that the adsorption energy calculation reflected the surface characteristics of the actual material.

## Conflict of Interest

The authors declare no conflict of interest.

## Supporting information



Supporting Information

## Data Availability

The data that support the findings of this study are available from the corresponding author upon reasonable request.
