# Supplementary material for: Establishment of “Structure‐Efficiency” Relationship in Ultra‐High Purity Metal Systems: Multi‐Scale Analysis of Tellurium as a Prototype
Source: Adv Sci (Weinh). 2025 Jul 29;12(40):e08531. doi: 10.1002/advs.202508531 (PMC12561281; doi:10.1002/advs.202508531)
Supplement: Supplementary file 1 — Supporting Information [file ADVS-12-e08531-s001.docx]

Supporting Information

**Establishment of “structure-efficiency” relationship in ultra-high purity metal systems: Multi-scale analysis of tellurium as a prototype**

*Shuai Guo^+^, Xianglei Dong^+^, Lin Zheng, Ming Gao, Guoqin Cao, Ping Peng, Jilin He, Junhua Hu^*^*

S. Guo, X. Dong, M. Gao, G. Cao, J. He, J. Hu

School of Materials Science and Engineering, Zhengzhou University

Zhengzhou 450001 (China)

E-mail: hujh@zzu.edu.cn

S. Guo, X. Dong, M. Gao, G. Cao, J. He, J. Hu

National Key Laboratory of Special Rare Metal Materials, Zhengzhou University

Zhengzhou 450001 (China)

L. Zheng

CNBM (Chengdu) Optoelectronic Materials CO., Ltd

Chengdu 610207 (China)

S. Guo, M. Gao, G. Cao, J. Hu

State Center for International Cooperation on Designer Low-Carbon & Environmental Materials (CDLCEM), Zhengzhou University

Zhengzhou 450001 (China)

M. Gao

School of Computational Science and Electronics, Hunan Institute of Engineering

Xiangtan 411104 (China)

P. Peng

School of Materials Science and Engineering, Hunan University

Changsha 410083 (China)

[^+^] These authors contributed equally to this work.


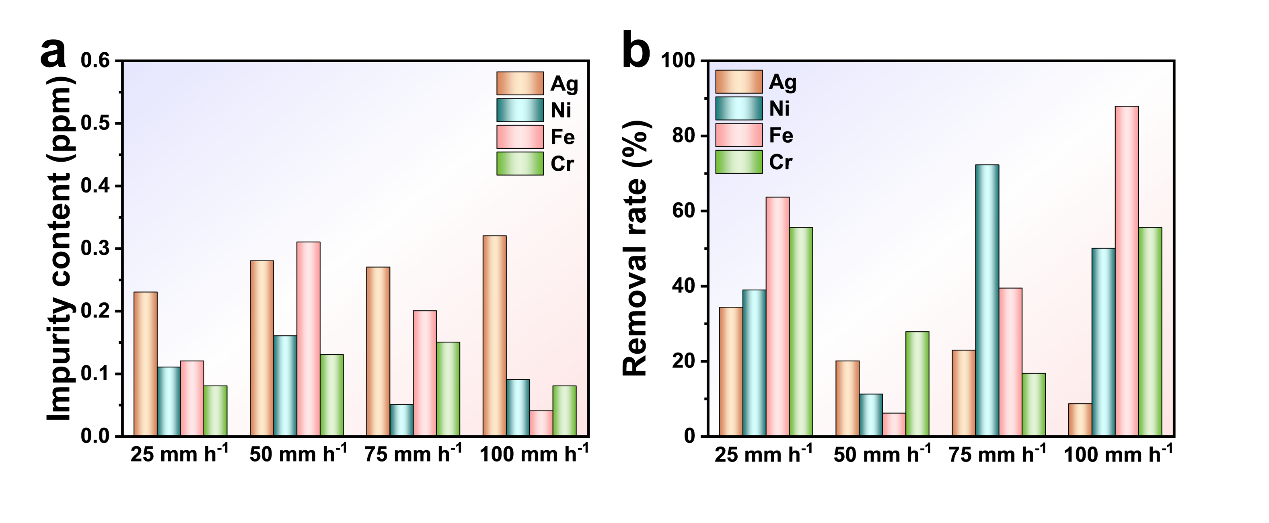


**Figure S1.** Impurity analysis of samples at different fusion rates. a) Impurity content. b) Impurity removal rate.

The effects of impurity separation can be summarized as follows: (1) Impurity enrichment driven by interfacial instability. Increased fusion rates can intensify undercooling and expand the mushy zone, inducing interfacial instabilities during solidification. This process gave rise to significant interface structure evolution, such as grain refinement or increased GBs density, which caused impurity elements to accumulate in the vicinity of the interface structure, making it difficult to effectively separate impurity elements. (2) Segregation kinetics governed by interface mobility. At sufficiently high fusion rates, the diffusion distance approached the interface thickness. Impurity atoms can be effectively captured by interfaces before they diffuse into the liquid phase, leading to impurity segregation deviating from equilibrium conditions and being driven by the interface structure related to kinetic motility. (3) Separation and purification mediated by interface anisotropy. In semi-metal purification systems, the liquid structure near the melting point differed significantly from that at higher temperatures, making the interface structure anisotropy more notable than in other systems. (4) Solid-phase adsorption hindering separation. The higher adsorption energy indicates that impurity atoms located at the solid-liquid interface tended to reject to the adsorption of the solid phase. The increase in fusion rate can lead to an increase in the adsorption energy of impurity atoms on the solid surface. However, the adsorption energies of elements such as Ag, Ni, Fe, and Cr were significantly high so that they were less constrained by adsorption from the solid phase, which induced the irregular change of content with the fusion rate. In summary, the above four factors are all related to the microstructure of the interface and are factors that influence impurity segregation. Changes in the surface interface microstructure caused by changes in the fusion rate can significantly affect the efficiency of impurity separation.


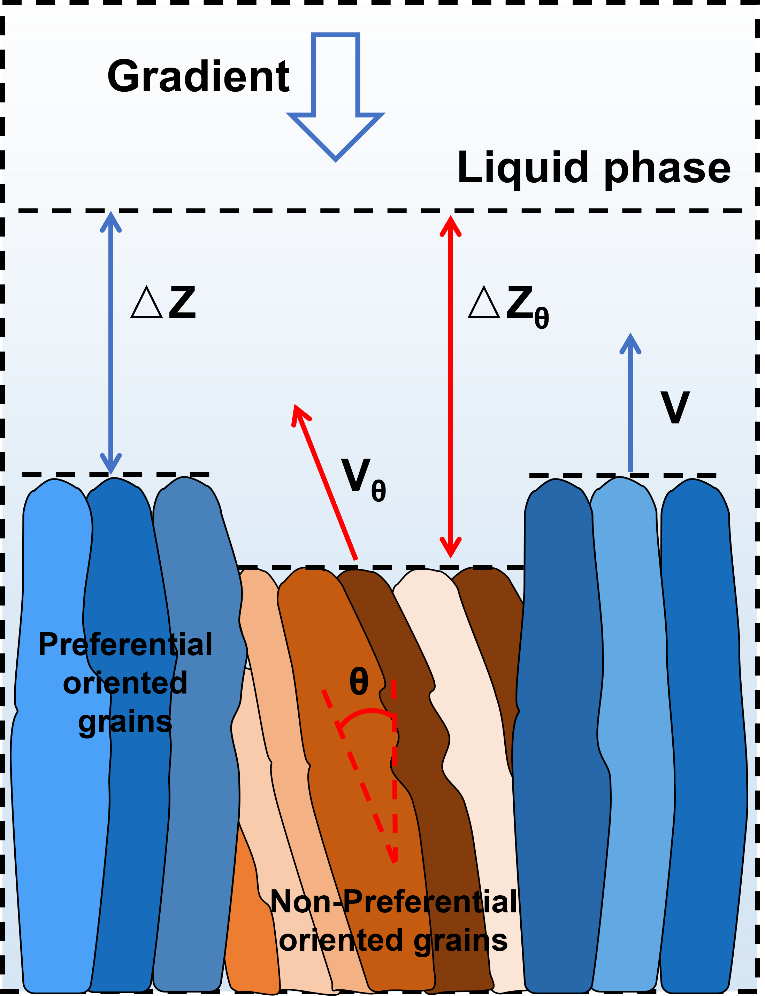


**Figure S2.** The competitive growth behavior of grains proposed by Walton–Chalmers model, which was governed by undercooling ahead of the solid-liquid interface.

**
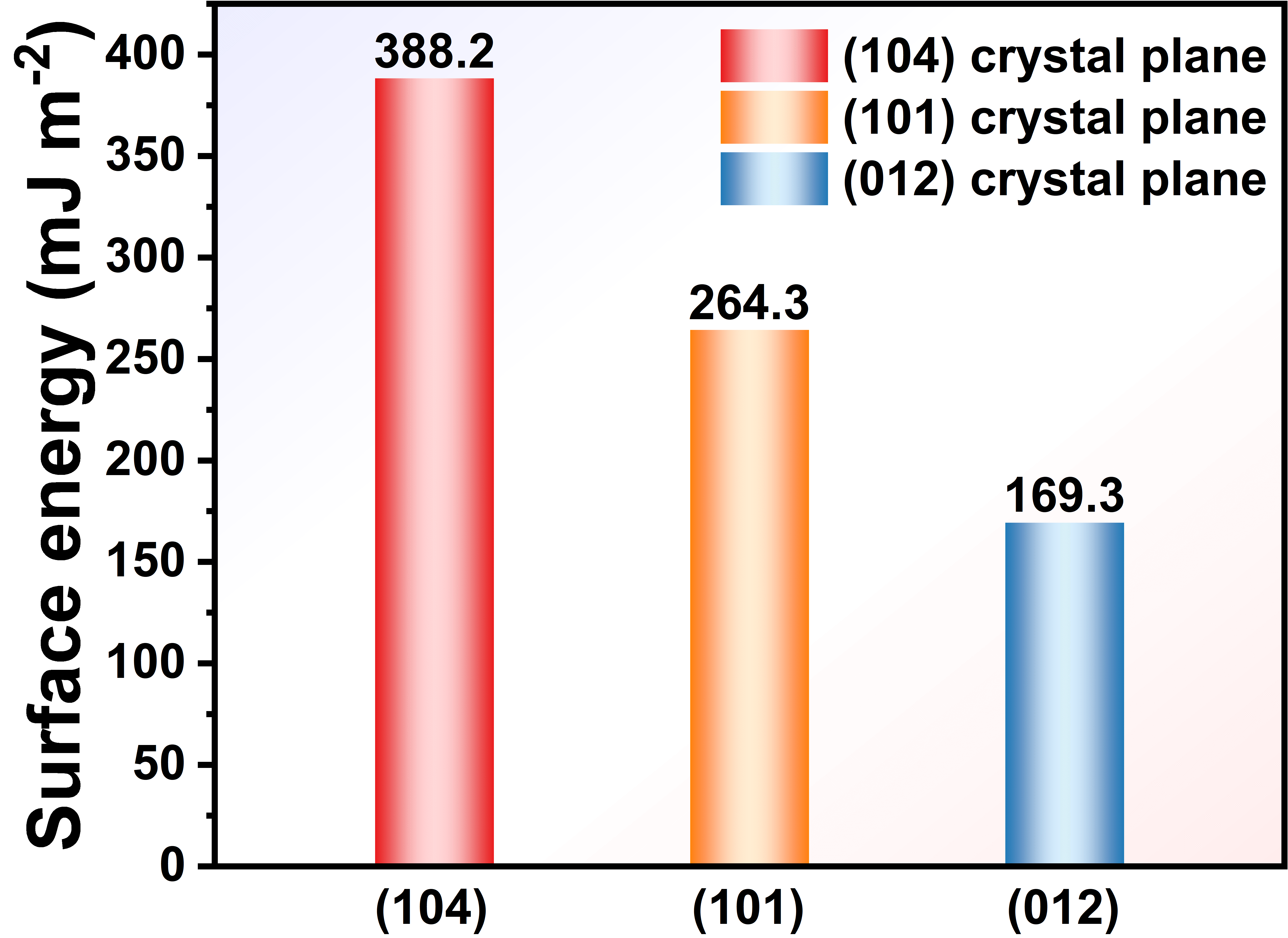
**

**Figure S3.** Surface energies of Te on (104), (101) and (012) crystal planes.

**
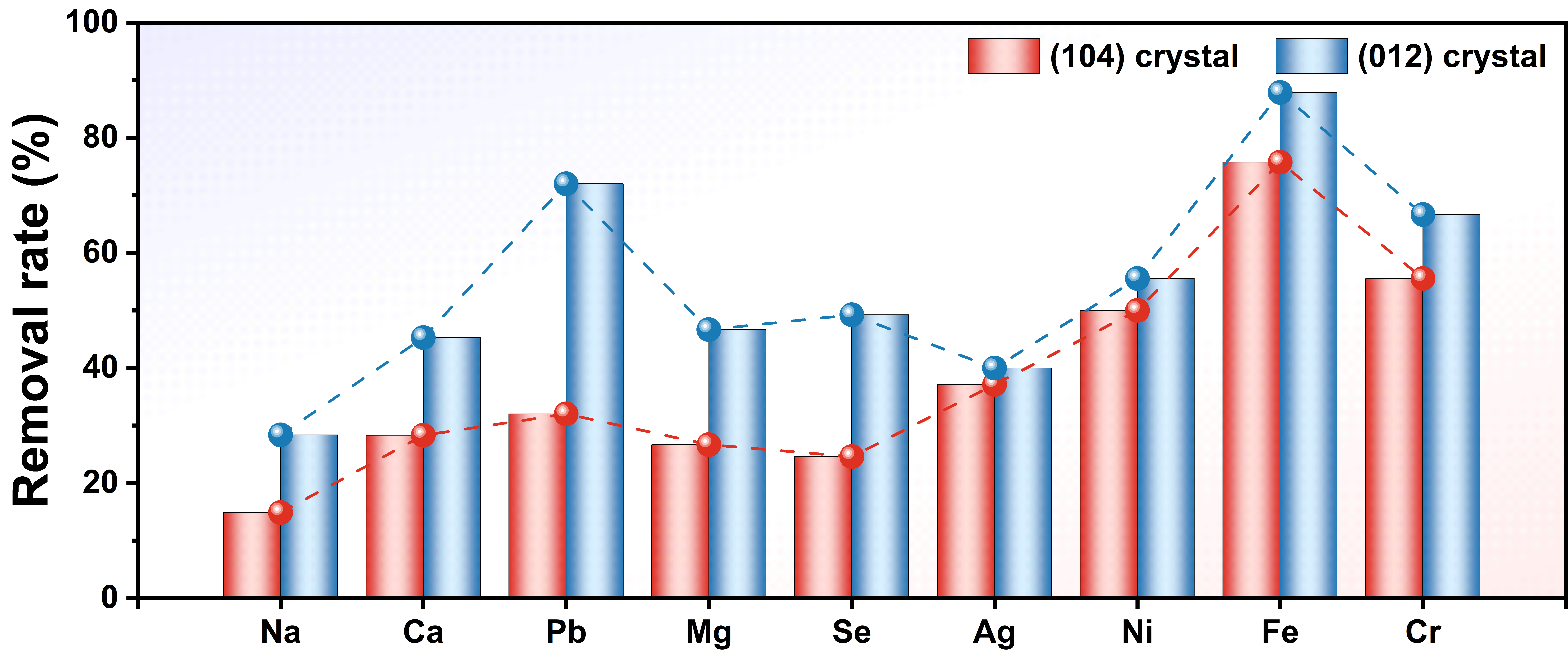
**

**Figure S4.** Impurity removal rate of Te crystals with (012) and (104) preferred orientation prepared by the Bridgman method.


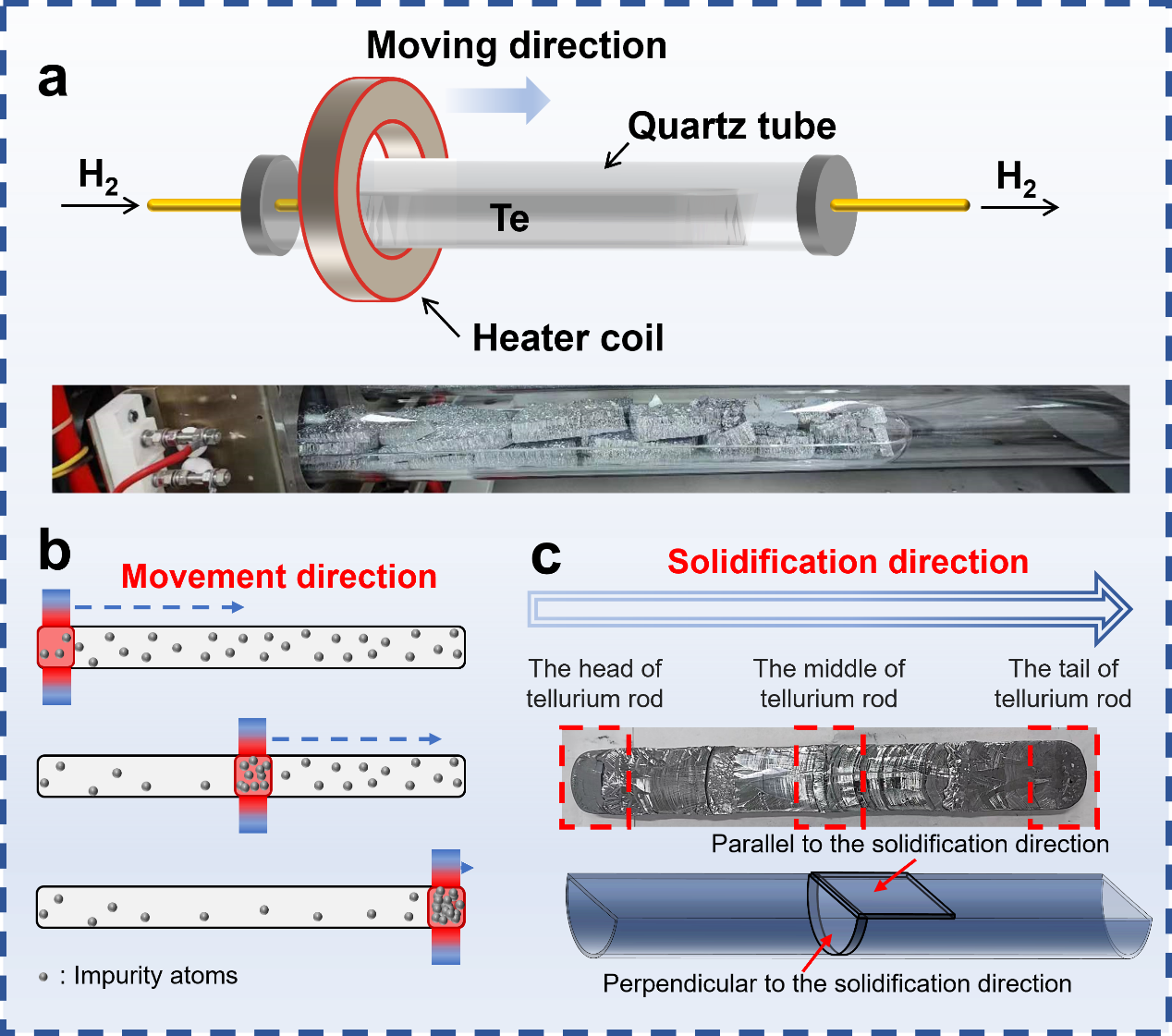


**Figure S5.** Schematic and analysis of zone refining. a) Schematic diagram of zone refining equipment. b) Schematic diagram of zone refining impurity atoms segregation. c) Sample diagram for zone refining.


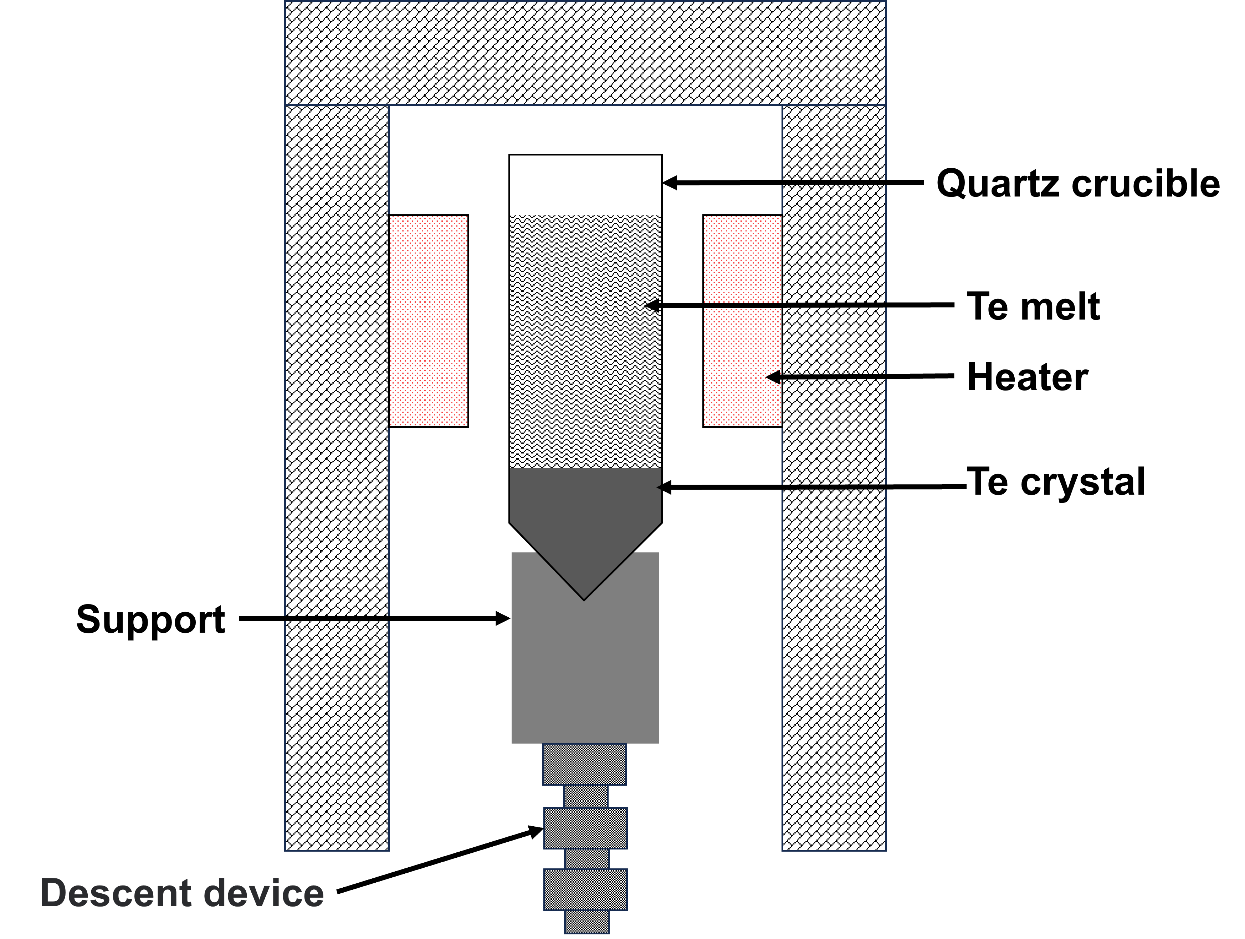


**Figure S6.** Schematic diagram of the vertical Bridgman method for Te crystal growth, comprising three main functional components: quartz crucible, heater, support, and descent device.

**Table S1.** Comparison of crystal plane strength ratio.

| **Sample** | **Crystal plane strength ratio** | **Ref.** |
| --- | --- | --- |
| Te-100 mm h^-1^ | *I_(012)_* / *I_(101)_*=9.52 | This work |
| Si | *I_(111)_* / *I_(220)_*=2.12 | [1] |
| Si | *I_(111)_* / *I_(220)_*=1.88 | [2] |
| Si | *I_(111)_* / *I_(220)_*=2.48 | [3] |
| Al-10%Zn | *I_(200)_* / *I_(111)_*=1.69 | [4] |

**Table S2.** Impurity content of raw Te

|  | **Impurity content** **(ppm)** | | | | | | | |
| --- | --- | --- | --- | --- | --- | --- | --- | --- |
| **Te** | **Na** | **Ca** | **Pb** | **Mg** | **Se** | **Ag** | **Ni** |  |
|  | 2.15 | 0.53 | 0.25 | 0.15 | 0.65 | 0.35 | 0.18 |  |
|  | **Fe** | **Cr** | **Cu** | **Zn** | **Cd** | **Al** | **Mn** |  |
|  | 0.33 | 0.18 | 0.01 | 0.01 | 0.01 | 0.01 | 0.01 |  |

**Table S3.** Comparison of the maximum fusion rate.

| **Sample** | **Maximum fusion rate** | **Ref.** |
| --- | --- | --- |
| Te | 100 mm h^-1^ | This work |
| Te | 6 mm h^-1^ | [5] |
| Te | 45 mm h^-1^ | [6] |
| Te | 30 mm h^-1^ | [7] |
| Te | 90 mm h^-1^ | [8] |
| Te | 60 mm h^-1^ | [9] |

We summarized the maximum fusion rate used in the literature on zone refining for the preparation of high-purity tellurium, as shown in **Table S3**. Most literature reports maximum fusion rates within the range of 6-90 mm h^-1^, so the fusion rates used in our experiment are reasonable. We have added the relevant content to the supplementary information and marked red.

# **References**

[1] Y. Zhu, J. Wu, K. Wei, W. Ma, Metall. Mater. Trans. B **2022**, 53 (4), 2704.

[2] L. Huang, J. Chen, A. Danaei, S. Thomas, L. Huang, X. Luo, M. Barati, J. Alloys Compd. **2018**, 734, 235.

[3] G. Qian, L. Zhou, S. Li, Z. Wang, L. Sun, ACS Sustainable Chem. Eng. **2021**, 9 (33), 11179.

[4] S. Shuai, X. Lin, Y. Dong, L. Hou, H. Liao, J. Wang, Z. Ren, J. Mater. Sci. Technol. **2019**, 35 (8), 1587.

[5] Q. Tian, Z. He, Z. Xu, D. Li, X. Guo, Metall. Mater. Trans. B **2024**, 55 (2), 772.

[6] M. Roumié, K. Zahraman, A. Zaiour, Y. Mohanna, M. Hage-Ali, J. Cryst. Growth **2006**, 289 (1), 260.

[7] N. R. Munirathnam, D. S. Prasad, C. Sudheer, T. L. Prakash, J. Cryst. Growth **2003**, 254 (1), 262.

[8] Q. Tian, Z. He, Z. Xu, X. Guo, Metall. Mater. Trans. B **2025**, 56 (2), 1709.

[9] D. S. Prasad, N. R. Munirathnam, J. V. Rao, T. L. Prakash, Mater. Lett. **2006**, 60 (15), 1875.
